# Fast ground irradiance computations for agrivoltaics via physics-informed deep learning models

**DOI:** 10.1038/s44172-025-00523-1

**Published:** 2025-10-07

**Authors:** L. Kurumundayil, D. Burkhardt, L. Gfüllner, S. J. Rupitsch, R. Preu, M. Berwind, M. Demant

**Affiliations:** 1 Fraunhofer Institute for Solar Energy Systems ISE, Freiburg, Germany; 2 IMTEK Department of Microsystems Engineering, University of Freiburg, Freiburg, Germany

**Keywords:** Computational science, Photovoltaics

## Abstract

Developing photovoltaic tracker algorithms for bifacial solar modules in agrivoltaic systems requires computationally intensive raytracing simulations to accurately quantify irradiation. Sunlight distribution on ground and module levels is essential for optimizing the setup and operation of tiltable PV systems, maximizing crop and electrical yield under various weather conditions and tilt configurations. We introduce a deep learning-based surrogate model that computes ground-level irradiation in a complex agrivoltaic scene with PV tracking. The surrogate model is physics-informed since the training data includes raytracing outputs based on real weather data. It computes the ground irradiance map based on direct normal irradiance, diffuse horizontal irradiance, solar position, and system geometry in just 3ms, four orders of magnitude faster than standard raytracing. The presented encoding of the 3D scene allows the calculation of ground irradiance using generative regression models. Our surrogate model allows on-the-fly raytracing calculations for edge computing-based PV tracker applications, where computational efforts must be minimized to enable efficient management and optimization of PV systems.

## Abstract (web summary)

Leslie Kurumundayil and colleagues present a deep learning-based surrogate model that computes ground-level irradiation maps for complex agrivoltaic systems. This model speeds up calculations, enabling efficient optimization of photovoltaic systems.

## Introduction

Agrivoltaics (APV) is the approach for dual use of land for agricultural purposes and photovoltaic (PV) electricity and is currently being implemented in many countries like Germany^1^, Japan^2^, Korea^3^, France^4^, and India^5^. In an APV system, simulations of ground irradiance describe the light conditions of crops planted below the photovoltaic panels. Excessive shading of the crops lead to decrease in agricultural yield while appropriate shading levels can increase agricultural yield^6^. Ground irradiance plays a vital role in the planning and construction of such systems, especially when considering the shading effects of substructures and PV module selection or positioning. The knowledge of the solar radiation distribution on the ground is not only crucial for the construction of the photovoltaic superstructure but also for the operation of tiltable PV modules, as well as for the crop selection, distribution, and cultivation. The knowledge of the ground solar irradiance can lead to substantial increase in energy yield and crop yield, since the optimum tracking position can be estimated correctly. For an operational APV system with 1-axis or 2-axis tracking, ground irradiance simulations can educate tracking algorithms that calculate the module tilt.

Irradiance values of scenes can be estimated using view-factor methods or ray tracing methods, which suffer from either reduced accuracy or long computation time. Standard PV simulation software such as PVsyst, pvPlanner using PVGIS, and SAM (System Advisor Model) are typically based on the computationally efficient and validated view-factor method but have limited functionality and validation for complex scenes. Raytracers like Radiance^7^ are known for their ability to produce physically accurate simulations of light behavior and propagation. However, the computations are time-consuming due to its complex algorithms and the large number of light paths that need to be traced. Render times can vary depending on the complexity of the scene and the desired image quality. To generate irradiance simulations using a raytracer, a desired number of virtual sensors are positioned across a virtual scene. The higher the number of sensors, the longer the computation time for a single irradiance map will be. For instance, to achieve a spatial resolution on the order of tens of centimeters for an APV scene with three rows of 40 modules, it can take at least 30 s to simulate a result using a raytracer.

For the successful operation and optimization of PV systems, multiple computer-expensive irradiance simulations are required. The number of simulations depends on the number of parameters that are taken into account for optimization. For instance, for an application requiring to compute hourly irradiance maps for a single crop season of 200 days with 50 varied tilt angles and 10 operating hours per day, it takes 10.000 simulations. For such applications requiring large temporal resolution, fast simulations of irradiance maps are essential. For each calculation, variations of the PV module tilt angle, Direct Normal Irradiance (DNI), Diffused Horizontal Irradiance (DHI), and the solar position must be considered, which leads to a high simulation effort.

Our work introduces a physics-informed deep learning model using for the fast computation of ground irradiance maps of agrivoltaic setups that takes the above mentioned parameters into account. This model acts as a surrogate for the raytracer to compute irradiance maps.

Numerous studies^8–10^ have employed deep learning methodologies within a forecasting framework to predict solar irradiance using historic weather data. Besides, there are research works^11^, that use deep learning approaches to predict the solar irradiance using astronomical and meteorological input parameters. In contrast to our work, these methods predict statistical values of the solar irradiance based on weather data only.

Our models are trained to predict irradiance map, taking into account both the meteorological data and the geometrical data of the PV module setup. This enables visualization of the shadow distribution on the ground. The geometrical configuration of a PV scene is complex, comprising multiple parameters, such as module height and tilt angle, resulting in a three-dimensional representation. Its complexity makes it unsuitable as an input for convolutional neural networks. In this work, we utilize the raytracer that generates top-view images of the PV plant, which encodes all the different parameters within itself, thereby encoding the 3-D information into 2-D maps. Machine learning methods such as lasso regression^12^ models and support vector regression^13^ are not applicable for this task due to complex 3-D geometries, and the inability to generate irradiance maps. Other generative methods, such as U-NET^14^ or diffusion models^15^, are successfully applied as surrogate models for the regression of physical quantities^16^. These models typically process two-dimensional input data, which is provided by our scene encoding.

As scientific contributions, we introduce a data-driven surrogate model (contribution 1) that inputs meteorological and geometrical parameters and predicts irradiance maps. We evaluate our approach by predicting corresponding ground irradiance maps for unknown seasons and unknown scene configurations, proving the generalizability of our approach to different APV layouts. For an efficient model training, we follow an unsupervised sampling strategy (contribution 2) to obtain relevant pairs of DNI, DHI, sun azimuth and elevation per month for network training. To encompass the complexity of 3-D geometries within a 2-D map, we use top-view images encoded with various parameters (contribution 3) as input to the model. For every pair of weather data, an irradiance map of the agrivoltaic scene at Morschenich-Alt was simulated using the software bifacial_radiance^17^ with customized functions to generate a paired dataset (contribution 4) for a successful model training. We run different training schemes to find the best possible surrogate model by varying the input parameters and using different combined loss mechanisms for model optimization.

**Fig. 1:**
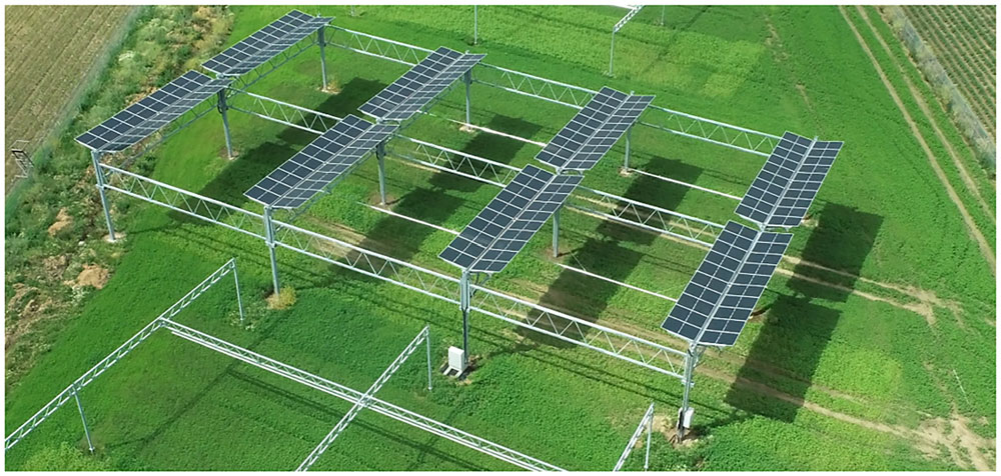
Drone image of the tracked agrivoltaic system in Morschenich-Alt. Photo taken by C. Kuchendorf, Forschungszentrum Jülich.

We evaluate our surrogate model for fast ground shadow simulations based on a real agrivoltaic system constructed in Morschenich-Alt, Germany, in cooperation with the Forschungszentrum Jülich^18^. A bird view of the system is shown in Fig. 1. It measures 44.4 m in length and 36.2 m in width. The system consists of four rows, each with 40 modules. The modules are placed six meters above ground with a pitch of twelve meters.

## Methods

### Overview of the pipeline

We introduce a machine learning pipeline for creating a surrogate model for raytracer simulations of the ground irradiance in an APV scene, which overcomes the challenge of long computation times. The surrogate model is trained via supervised learning. The input data comprises the weather data and scene geometry and the output data are the corresponding ground irradiance maps. Despite the common architecture of the model, we have to tackle several challenges for an efficient and successful model training.

**Fig. 2:**
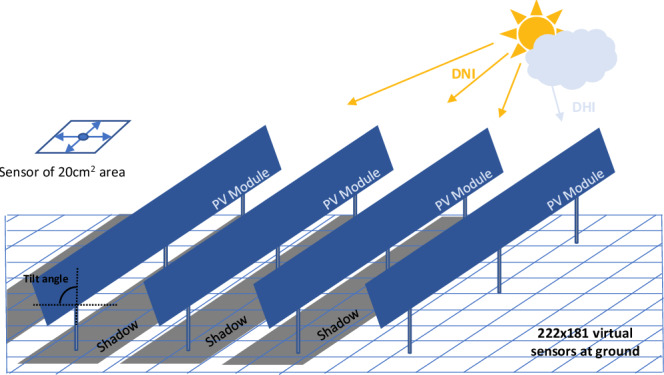
A schematic depicting the real agrivoltaic system based in Jülich, Germany. Sensors are virtually placed on the ground across the agrivoltaic scene leading to simulated irradiance values given weather information including direct normal irradiance (DNI) and diffused horizontal irradiance (DHI).

1. For the training of a generalized model, a comprehensive dataset is required. To guarantee that we train our model with relevant meteorological data and to reduce computational effort in generating raytracer simulations, we use a special sampling of the weather data based on clustering algorithms of historic data.
2. The ground shadow does not only vary based on the meteorological data but also on the geometry of the APV system. The simulated irradiance maps depend for a complex scene description, which is not an applicable input for a deep learning model. Therefore, we have to encode the scene geometry.
3. The surrogate model uses a fully convolutional neural network for the regression task and learns to generate the ground shadow irradiance map from the inputs. Generative models which are trained with a pixel-wise loss tend to generate unsharp images^19^. We combine the pixel-wise loss with an adversarial loss^20^ to encourage the generation of high-frequency details.
4. For the experimental evaluation, we consider a single APV system layout with varying module tilt angles, making the ground irradiance map susceptible to the module tilt angle. Figure 2 shows a schematic of the APV system for which the data is simulated in our work. We evaluate the generalizability to other data, by evaluating crop seasons not considered during training, and APV layouts with varying pitch, height, and tilt angle shown in Supplementary Note 1.

### Sampling strategy for meteorological data

For our dataset creation, we use historical data from the years 2010 to 2019, which were downloaded from the DWD^21^ open database. The data contains the global horizontal irradiance (GHI) and DNI. GHI is the radiation received by a horizontal surface and consists of DNI and DHI, as formulated in Eq. (1). DNI is the amount of radiation received per unit area of a surface perpendicular to the sun. DHI is the radiation, that is scattered in the atmosphere, received by a horizontal surface.

1 $${{{\rm{GHI}}}}={{{\rm{DNI}}}}\cdot cos\theta +{{{\rm{DHI}}}}$$

**Fig. 3:**
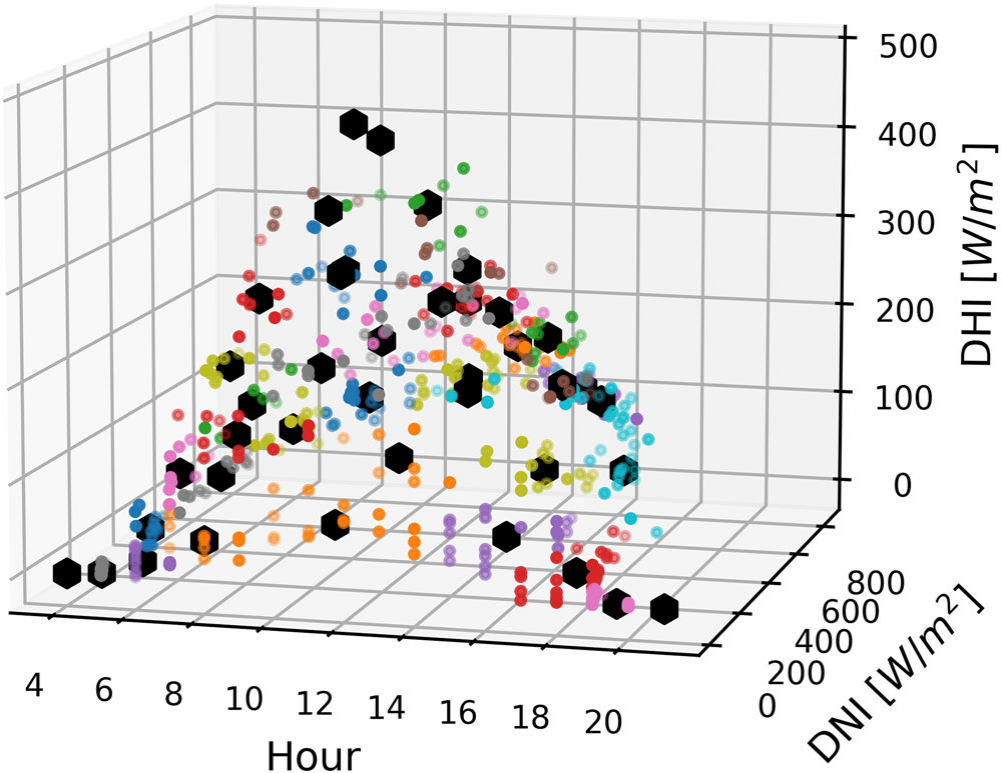
3D visualization of meteorological data from 2010 to 2019. The data is clustered into 40 groups using k-means clustering algorithm. Every datapoint is color-coded to its cluster.

*θ* is the angle of the incident beam. All parameters are measured in W m^−2^. To feed our network with suitable meteorological data, we use a unique sampling method to find relevant pairs of DHI, DNI, sun azimuth and sun elevation. Using the historic weather data from recent years, a thorough analysis was conducted to find the best sampling points of the different weather parameters for each hour. In order to sample through the different parameter values, a k-means clustering ^22,23^ was used. The solar position comprising of the azimuth and the elevation parameters for every hour is obtained using the python package pvlib^24^. The weather data of each year are separated into months. Using the k-means clustering algorithm, the data is clustered into different groups based on the DHI, DNI and solar positions. The data is split into 40 clusters, resulting in at least one sampling point at every hour between 05:00 and 20:00. Figure 3 shows the 3D visualization of the clusters, obtained by the clustering algorithm for the month of August. The x-axis shows every hour during daytime, i.e. 4 a.m. to 8 p.m. The y-axis shows the range of DNI in W m^−2^, whereas the z-axis shows the range of DHI in W m^−2^. Each plotted datapoint is color-coded according to the cluster it belongs to. The large hexagon-shaped black dots indicate the cluster centers. As shown, the cluster centers are well distributed over the weather data which indicates a representative sampling, allowing an efficient creation of training data.

### Encoding of the scene geometry

For our network to learn the shadow distribution on the ground accurately, it is crucial to feed the network with the geometry of the agrivoltaic system. The geometry of an APV system consists of multiple parameters: tilt angle, number of rows, number of modules per row, pitch, etc. which are encoded successfully within our approach.

By only providing these parameters as a scalar value, the input data presumably lack information about the remaining details of the scene geometry, which is evaluated in Results for tilt variations.

To include all geometrical information and the spatial relations of the objects, we generated top-view depth images of the scene using the depth buffer of the Radiance raytracer. The geometrical parameters and relations of the APV system are encoded in this input map. Additionally, every pixel gives the distance to the virtual source camera. To record the depth images, the camera is virtually placed 40 units above ground. Therefore, the ground has a value of 40 (deepest point), whereas the modules have varying depth values depending on the tilt angle.

In our first evaluation, we evaluate a single-axis tracking layout with fixed parameters except the module tilt angle. For data generation, we sample through the module tilt angles in the range of [−40°, 40°] with a step size of two degrees. The tilt angle is required as input for the scene geometry. In our second evaluation, we also vary further geometrical parameters: tilt angle in range of [−40°, 40°], pitch in range of [6 m, 12 m] and height in range of [2.5 m, 5.5 m].

### Raytracer simulations

The raytracer Radiance was exploited to generate the scene geometry and simulate the ground irradiance maps via the python package bifacial_radiance^17^. To achieve a high spatial resolution of 20 cm in both x- and y- direction during rendering, we virtually place a total of 181 sensors along the width and 222 sensors along the length of the APV scene, resulting in a total of 40,182 sensors on the ground. A customized EPW weather file, containing hourly weather parameters, is required to simulate irradiance maps. We create such a weather file using the resulting cluster centers from the above mentioned sampling scheme. The sensors are virtually placed in the rendered scene, and a raytracer simulation is computed depending on the sun’s position, and diffused and direct sunlight. The raytracer package Accelerad^25^ was used for simulations to reduce the high computation time, due to the high number of sensors.

### Surrogate model: design and optimization

**Fig. 4:**
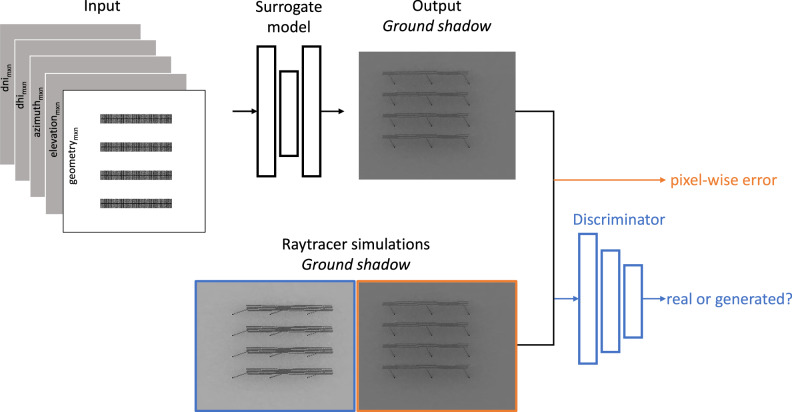
Training scheme of the surrogate model. Given a 5-channel input, the model learns to predict the ground irradiance map by optimizing via a combined pixel-wise and adversarial loss function.

The training scheme of our surrogate model is shown in Fig. 4. We utilize a fully convolutional neural network based on the U-Net^14^ architecture, with the final layers modified to enable our regression task. This design enables the network to identify complex patterns in images and process data with fewer parameters, while ensuring rapid output computation. The model processes a 5-channel input that comprises of weather data (DNI, DHI, azimuth and elevation) and scene geometry encoded as top view image to compute a ground shadow map.

In detail, it consists of four downsampling and four upsampling steps, each comprising two 3 × 3 convolutional layers followed by Leaky ReLU activation. Each downsampling step is concluded with a 2 × 2 max pooling layer, while each upsampling step ends with a 2 × 2 transposed convolution layer. At the bottleneck, an additional block of two 3 × 3 convolutional layers and two Leaky ReLU layers is applied. Crucially, skip connections are implemented to link the corresponding encoder and decoder layers. The final layer is a 1 × 1 convolution layer. Further details are listed in Supplementary Note 2 including the model architecture in Supplementary Fig. 3.

Our model is optimized using a combined loss mechanism of a pixel-wise loss and an adversarial loss. The pixel-wise error is estimated between the generated ground shadow (output) and their respective raytracer simulation used as reference. To obtain the adversarial loss, another neural network called Discriminator is trained on both generated ground shadow maps and raytracer simulations. Given the generated output, the discriminator predicts whether the output is generated or a raytracer-simulated image. The adversarial loss aids in generating indistinguishable outputs by improving the realism within the generated outputs. During stochastic gradient descent, errors are back-propagated through the network and the network weights are updated using the Adam optimizer^26^. A learning rate scheduler with gamma = 0.2 was employed for all the experiments.

### Experiments

At first, we conduct five experiments to evaluate the quality of the surrogate model with two possibilities of feeding the scene geometry and varying loss mechanisms to identify the best model configuration. Within an ablation study, we investigate the impact of different components added to or removed from our model. The design of experiment is shown in Table 1. In a second step, the generalizability of the model on varying scene geometries is evaluated and presented in detail in Supplementary Note 1.

**Table 1:** Overview of experiments conducted to obtain the best model weights

Experiment	Input		Output	Optimization	
	Scene input	Variation	Resolution	*ℓ*_1_loss	Discriminator loss
Supervised_TiltAngle	Scalar	Tilt angle	20 cm	1	0
Supervised_TopView	Depth image	Tilt angle	20 cm	1	0
Unsupervised_TopView	Depth image	Tilt angle	20 cm	0	1
Mixed_balanced	Depth image	Tilt angle	20 cm	1	1
Mixed_optimized	Depth image	Tilt angle	20 cm	1	0.7
Generalizability_TopView	Depth image	Tilt angle pitch height	20 cm	1	0

#### Training and test split

The dataset, based on sampled weather data from the years 2010 to 2019, is divided into training and validation set of 8000 and 2000 samples, respectively. To test our models on unseen data, we generated an additional dataset with meteorological data collected for the year 2021.

#### Depth image as input

We analyze two ways of encoding the scene configuration: encoding the scene geometry as (1) a scalar value of the tilt angle (Supervised_TiltAngle) and as (2) a spatially resolved input (remaining experiments) given by the depth map of the scene which is rendered from the top. For the latter, a depth map can be precomputed once for every possible tilt angle since it does not depend on the meteorological data.

#### Supervised and unsupervised optimization

We investigate the effect of different loss mechanisms when optimizing the models. In the Supervised_TopView and Supervised_TiltValue experiments, the models are optimized by minimizing the mean absolute error, also called *ℓ*_1_-loss, between the predicted and the target ground irradiance map. The *ℓ*_1_-loss is the average of the absolute differences between the predicted and the target values. In the Unsupervised_TopView experiment, we analyze the use of an adversarial loss function. The Mixed_balanced and Mixed_optimized experiments combine both loss functions with different weighting strategies. To train a model via adversarial learning, a second network, namely discriminator, is trained to reveal if an image has been generated or simulated and thus provides feedback to the generator. The discriminator can be trained with historic generated data and randomly selected simulated data and is updated from time to time to enhance accordingly. During training of the generator, a ground irradiance map is passed through the discriminator. If the discriminator distinguishes the predicted irradiance map from the raytracer simulation, the generator will update its weights to improve performance.

#### Generalizability

In the second step of our experimental evaluation, we consider the generalizability of model, by taking into account varying geometries of the APV layout. The data generation and results of the generalized model are found in Supplementary Note 1.

## Results

### Computation time

We compare the computation time of the raytracers Radiance and Accelerad with the surrogate model for different resolutions. The reported computation time include initialization, simulation and the saving. At a resolution of 20 cm, which corresponds to 40,182 sensors, Radiance completes a simulation within 33 s, whereas Accelerad takes 10 s. By increasing the resolution to a sampling distance of 10 cm, thereby increasing the number of sensors, Radiance and Accelerad require 109 s and 86 s, respectively. The surrogate model, trained on either low resolution or high resolution simulations, can generate a ground shadow in just 3 milliseconds on a GPU, and therefore outperforming the raytracer for both resolutions.

### Prediction of irradiance maps

**Fig. 5:**
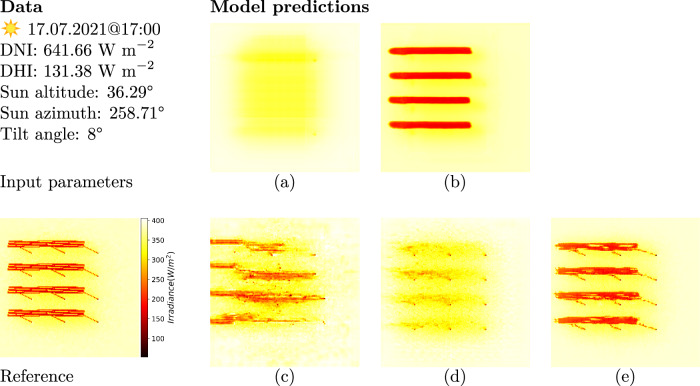
Overview of model predictions from different experimental setups. The first column shows the input parameters and the reference image. The remaining columns show the results from **a** Supervised_TiltAngle, **b** Supervised_TopView, **c** Unsupervised_TopView, **d** Mixed_balanced, and **e** Mixed_optimized.

The surrogate model predicts the ground shadow maps for any given pair of weather and geometry parameters. We trained and validated our model in five experiments described in Methods to identify the best model configuration and optimization. We evaluated each model for unknown crop seasons, as shown in Fig. 5. The results on generalizability for different scene configurations are shown in Supplementary Note 1.

The first column shows the input and reference data in the first and second rows respectively. The input consists of the DNI, DHI and the sun’s position (altitude and azimuth), and the module tilt angle. The reference data is the respective raytracer-simulated image for the given input parameters. The second column shows the model predictions for each conducted experiment. The model predictions in the first row show the results of models trained using a supervised loss mechanism.

#### Scene encoding

Figure 5a shows the result generated with supervised learning (Supervised_TiltAngle). In this experiment, the model takes the module tilt angle as a scalar input rather than a spatial input. However, the model does not predict the shadow at all using the module tilt angle as the only geometrical input. Figure 5b shows the results generated in Supervised_TopView experiment. The model has fairly succeeded in computing shadows respective to the weather data, and is capable to produce the shadows, cast by the modules, accurately like in the reference image. It can be observed that details at the pole shadow are neglected.

#### Comparison of losses

The second row shows the results obtained from models trained using different combinations of losses with pixel-wise loss and adversarial loss. Figure 5c depitcs the output of the model trained in a fully unsupervised manner (Unsupervised_TopView) using the GAN only. Figure 5d, e displays the results of the models trained in Mixed_balanced and Mixed_optimized experiments. Using a strong discriminator loss failed to create more realistic-looking model predictions in the Unsupervised_TopView and Mixed_balanced experiments. The models were not able to predict the ground shadow at all due to a phenomenon known as mode collapse^27,28^. The generator learns to generator a single realistic example which fools the discriminator. However, the model trained in Mixed_optimized experiment created a ground irradiance map which is similar to the ground truth data, including the shadows cast by pole structures.

#### Weather scenarios

**Fig. 6:**
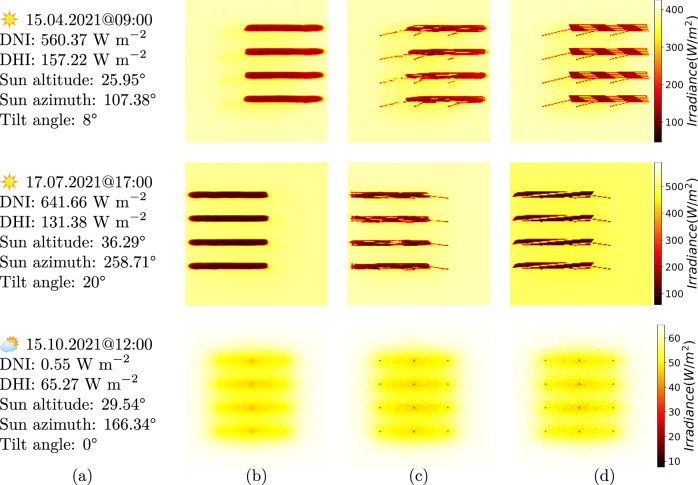
Results for different weather scenarios in each row. Column (**a**) shows the input data. The columns (**b**) and **c** show the predicted irradance maps without (Supvervised_TopView) and with adversarial loss (Mixed_optimized), respectively. For all scenarios, both models achieve a high similarity to the simulated irradiance maps shown in column (**d**).

Figure 6 shows the results obtained from the surrogate model trained with top view images as input for three different weather scenarios: Spring morning, Summer afternoon and Autumn noon. In all three examples, the first image depicts the predicted ground shadow map and the second image shows the corresponding raytracer simulation for reference. The first example shows the model prediction for a spring morning with high direct irradiance. The second example depicts a sunny afternoon at 5 p.m. with an even higher direct irradiance. The third example shows the results for a autumn noon with very low direct irradiance and low diffused irradiance. The predicted images display similar values as their corresponding irradiance maps. For the spring evening and both summer examples, the surrogate model predicts the module shadows at the right position on the ground and also learns to predict a more consistent shadow than the actual target images. When modeling an autumn day, the diffuse light will increase the irradiance in the shaded region accurately.

### Application

#### Determine irradiance development for individual crops over time

**Fig. 7:**
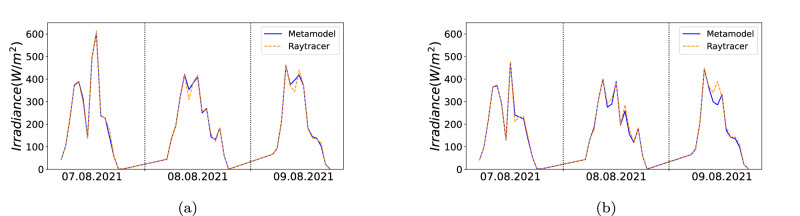
3-day irradiance calculation with surrogate model and raytracer simulation. Comparison between surrogate model predictions an raytracer simulations for **a** a fixed tilt PV system and **b** a tracked PV system.

Many applications in precision farming, e.g. water management, require an accurate quantification of the irradiance at crop level. The surrogate model can provide spatially resolved irradiance maps for a longer period of time with high accuracy. To demonstrate this, we select a spot on the ground and predict the irradiance and compare it to the same spot in the real raytracer simulations. The sensitivity of the model with regard to the tilt angle is shown by comparing a system with fixed tilt and a sun-tracking system. Figure 7 shows the comparison between the predicted and raytraced irradiance values for the days, 7^th^ to 9^th^ August 2021 excluding the night hours. Figure 7a depicts the plot for a fixed-angle system with a module tilt angle of 30°, whereas Figure 7b shows the plot for a sun-tracked APV system. Both plots demonstrate that the surrogate model accurately predicts the irradiance values and closely follows the curve observed in the ray-traced simulation, with only minor differences. The resulting differences are due to two reasons: (1) the raytracer simulated images exhibit artifacts due to the aliasing effect, which do not appear in the surrogate model predictions, and (2) the surrogate model’s uncertainty around the module edges.

#### Accumulated light distribution for crop planning

**Fig. 8:**
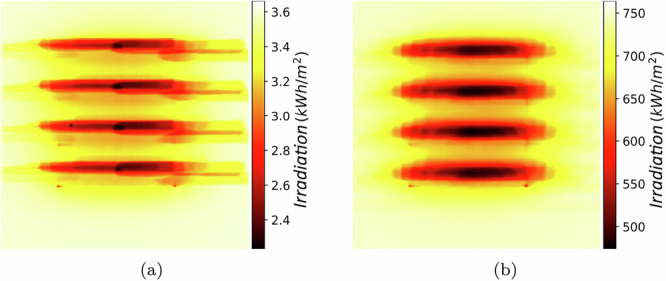
Maps of accumulated irradiation distribution. **a** Distribution over a single day in April. **b** Distribution over the full crop season from April to October.

In this application, we illustrate how our surrogate model can be used to look into accumulated ground light distributions for a certain period of time. Figure 8 shows two examples of accumulated ground light distributions as heatmaps. These heatmaps reveal the light distribution within a PV-mounted field during a given period of time. Such heatmaps could allow an efficient planning of an APV construction. We have computed a heatmap to demonstrate the daily sunlight distributions. Figure 8a shows the heatmap for the accumulated irradiation distributions on a spring day in April. The sunlight for a spring day follows a distribution between 2.4 kWh m^−2^ day^−1^ and 3.6 kWh m^−2^ day^−1^. Furthermore, Fig. 8b shows the seasonal light distribution map for a season from April 2020 to October 2020. The ground below the modules has the least light distributions of around 500 kWh m^−2^ season^−1^ whereas the surrounding areas have higher light distributions of about 700 kWh m^−2^ season^−1^. It is important to note that it takes only about 5 minutes to generate such a heatmap. This enables a flexible and efficient planning and management of APV systems.

## Discussion

### Encoding of the scene geometry

First, we analyzed the importance of having a top-view tilt angle-encoded depth image. From the model predictions in Fig. 5a, b, we observed that it is challenging for the model to learn the patterns without additional knowledge about the scene. Including more geometrical parameters as model inputs increases complexity and computational costs. However, using a geometry-encoded depth image enabled the prediction of irradiance maps shown in Fig. 5.

The presented approach for scene encoding can be used for various computation tasks relevant for the operation of any sun-tracked PV power plants. Placing the sensor data of the raytracer simulation onto the front and backside of the PV modules enables the estimation of irradiance on the device. Training the surrogate model with module-level irradiance values allows for a fast computation of irradiance maps and thus for an accurate PV yield prediction for each module.

### Loss mechanisms

Among all the experiments shown in Fig. 5, the models in Supervised_TopView and Mixed_optimized experiments demonstrated promising results. They successfully recreated the shadows projected by the modules at the correct positions given weather and geometry inputs. However, the Supervised_TopView model, unlike the Mixed_optimized model, could not generate shadows projected by the vertical pole structures. This is due to the absence of information about the pole structures in the input data. The top-view tilt angle-encoded image used as the geometry input does not provide insight into the pole structures, which are only visible from a side point of view.

The combined loss mechanism of *ℓ*_1_ loss and adversarial loss was able to create the shadow of the pole structures. Models optimized with adversarial loss only were unable to generate relevant ground shadows due to mode collapse, where the model predicts only a certain type of output. When the discriminator loss was tuned down to 70% compared to the *ℓ*_1_ loss, the model would produce realistic shadows similar to the reference image, mitigating mode collapse. The advantage of this model is that it learns to produce shadows cast by the pole structures. Yet, it is prone to the aliasing effect from the raytracer simulations learned through the discriminator loss, which can be addressed in future works.

We conclude that the Supervised_TopView and Mixed_optimized models trained on simulations are the best models. The absence of pole shadows on the ground in the Supervised_TopView model-predicted maps is not relevant as it does not impact the overall shadow caused by the modules. Interestingly, we can observe that simulation artifacts like aliasing and grain noise are smoothed out within the surrogate model. Although they represent as differences to the raytracer simulations, these artifacts do not impact the resulting ground irradiance calculations. However, the Mixed_optimized model can be used if the pole structures are relevant.

### Aliasing artifacts

Analysis of the raytracer simulations revealed artifacts within the module structures caused by aliasing as shown in Fig. 6. These artifacts are omitted by the supervised model, but is visible when using adversarial models. Increasing the sensor resolution of the raytracer simulation avoids this issue. However, the simulation time increases to 80 s per simulation by a factor of four. This will increase the training time of the surrogate model, but further leverage the benefit in computation time when applying the model. Another potential approach to remove aliasing artifacts from simulated images is NVIDIA’s DLSS 2.0^29^, which uses a convolutional autoencoder to predict high-resolution anti-aliased images from low-resolution aliased images and their motion vectors, generated by a game engine. However, based on our results, we believe that lower resolution simulations are sufficient to train an efficient surrogate model.

### Generalizability

Generalizability_TopView experiment was carried out to address the generalizability of our surrogate model to varying geometries of the PV systems for ground irradiance predictions. Please refer to Supplementary Note 1 for the detailed results. Our model was successfully trained on an extended dataset with varying parameters and tested with unkown geometries. The surrogate model demonstrates generalizability. It effectively generates irradiance maps for inputs with varying row pitches, module heights and module tilt angles, and thus can be applied to the use of more traditional utility scale systems. It could be difficult for other types of PV systems like vertically mounted modules, as the scene encoding becomes more challenging for such systems.

### Outlook

The surrogate model is useful for time- and computationally expensive PV and crop yield calculations. Traditional PV simulations are not computationally intensive apart from light calculations via a full ray-tracer or even view factor methods. However, our integrated PV use case requires extra computational effort for these light calculations: spatio-temporal heterogeneous irradiation must additionally be calculated on the ground layer to properly analyze agrivoltaics, which costs computational resources. Irradiance maps are also relevant for the PV-use case, when self-shading and diffuse irradiance affect PV-yield. The irradiance distribution for different tilt sequences is a fundamental requirement for tracking optimization, since irradiation typically linearly correlates to PV yield.

**Fig. 9:**
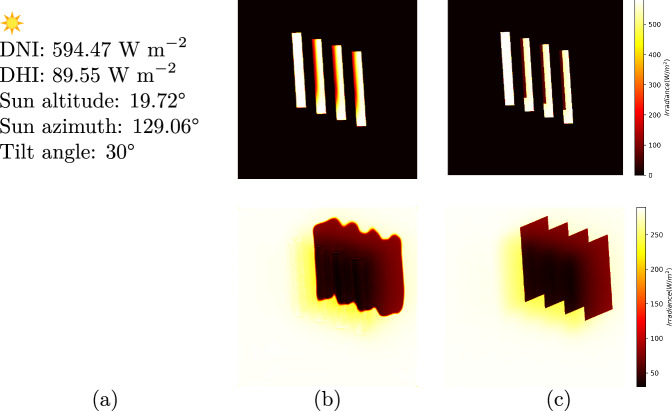
Results for sensors placed at different levels in an APV scene. The first row shows the irradiance maps for sensor data placed at module level, while the second row shows the maps for sensors placed at ground level. The weather data is shown in column (**a**). Columns (**b**) and **c** show the predicted irradiance maps and the reference raytracer simulations, respectively.

For annual PV yield prediction the surrogate model can be adjusted: by including sensors at module-level, the model training can be extended to predict module-level irradiance maps. Figure 9 shows first results of a surrogate model trained on an extended dataset with sensors placed on the module for a sunny hour. The input parameters for said example are provided in Fig. 9a. The first row shows the results for the top-side of the modules, whereas the second row shows the results for the ground-level irradiances. Figure 9b, c shows the model predictions and the reference raytracer simulations, respectively. Here, the self-shading effect is observed, which occurs when the shadow cast by a module falls onto the neighboring module. This furthermore demonstrates the proof-of-concept of our work, and allows for an accurate PV yield analysis, which will be evaluated in future works.

## Conclusion

We demonstrated a machine learning pipeline to train a deep learning model for the generation of irradiance maps based on meteorological and scene geometry input data. By encoding the complex scene geometry within a top-view image, we are able to process the 3D scene within a convolutional neural network for the regression task. To achieve this, we trained our model on carefully curated data, employing a unique sampling method to capture a representative set of grouped data of DNI, DHI and solar position, which includes sun azimuth and sun altitude. For each combination of meteorological parameters and module tilt angles, an irradiance map is simulated using a raytracer to create paired data.

We conducted six different experiments, varying the scene input encoding and loss functions, to identify the optimal model. The best model was trained with the proposed top-view scene input encoding with a combination of pixel-wise *ℓ*_1_ loss and adversarial loss. The latter allows to reconstruct high-frequent structures like poles of the APV plant. This model accurately generates shadows at scene locations for the given weather parameters. Using CNNs to generate ground shadow maps drastically reduces computation time to 3 ms compared to ~30 s for raytracer simulations.

Our surrogate model enables fast computation of irradiance maps which can be exploited in various applications like APV/PV systems construction, forecasting and operation. The model has been evaluated on ground irradiance data. Adding sensors to the front and backside of the PV modules will enable to predict irradiance maps of the PV modules and thus allow to estimate the PV yield.

## Supplementary information

Supplementary Information

## Data Availability

The data that support the findings of this study are openly available in Figshare at 10.6084/m9.figshare.27004783.

## Code availability

The code used for model training and inference and plotting cannot be made public, as this work is part of a confidential project. However, a description of the methods and algorithms used is provided within the manuscript.
