# Supplementary material for: Fast ground irradiance computations for agrivoltaics via physics-informed deep learning models
Source: Commun Eng. 2025 Oct 7;4:173. doi: 10.1038/s44172-025-00523-1 (PMC12504610; doi:10.1038/s44172-025-00523-1)
Supplement: Supplementary file 1 — Supplementary Information [file 44172_2025_523_MOESM1_ESM.pdf]

Supplementary Note 1

Evaluation of varying photovoltaic layouts

Data generation

Additional experiments were carried out to address the generalizability of the surrogate model introduced in Methods on varying photovoltaic layouts.

We trained the model on an extended dataset that includes varying geometries for photovoltaic layouts. The dataset comprises weather and geometry data as the input, and its respective raytracer simulations as the label. The geometries are adapted to incorporate more variation between three parameters: (a) tilt angle in the range of [-40 °,40 °], (ii) pitch in the range of [6m, 12m], and (iii) height of the mounted modules in the range of [2.5m, 6.5m]. We applied a random sampling strategy among these three parameters to generate the dataset. Using strategically sampled weather data from historical weather data, explained in Methods, and the newly sampled geometries with variation in tilt angle, pitch, and height, the ground irradiance maps are simulated using the Radiance raytracer.

The varying PV configurations are successfully encoded in the top view images. Figure 1 shows three examples with varying tilt angle, pitch, and height in . The variation in the tilt angle within the modules can be seen in each image. Figure 1(a) and Figure 1(b) shows how the values change for arbitrary tilt angles, whereas Figure 1(c) shows that the height values do not change for 0° tilted modules. Also, the variation in the pitch between rows is encoded in the top-view images. The height of the modules is encoded in the absolute values of the modules. For visualization purposes, the images, which are originally scaled between 0 and 40 units, are inverted in scale.

Training, validation and test data. The training and validation datasets are based on weather data from years 2010-2019, resulting in a dataset of about 11000 datapoints, of which 80% is used for training and 20% is used for validation. For testing purpose, new data based on geometries that are not within the training and validation datasets, are generated. The resulting test dataset contains data based on the 2021 weather data.

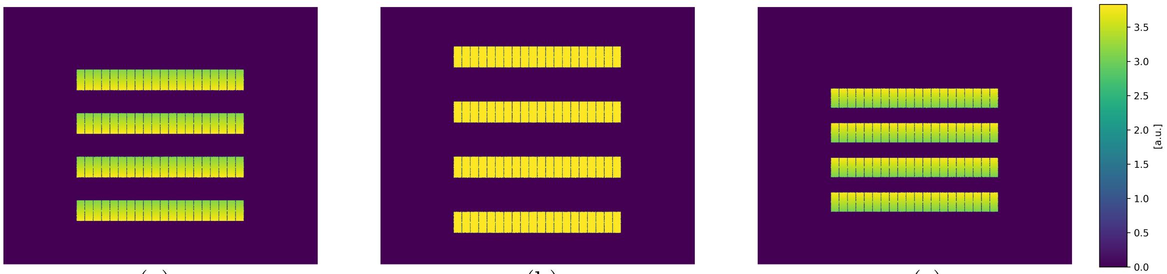

(a) (b) (c)

Supplementary Figure 1: Top-view images for different geometries. (a) tilt angle = -10.3°, pitch=8.3m, height=2.5m; (b) tilt angle = 0.0°, pitch=10.5m, height=5.6m; (c) tilt angle = 23.5°, pitch=6.6m, height=5.7m

Results

The surrogate model was trained and validated with the newly generated dataset, and successfully converged. Figure 2 shows the results of the model tested on the unseen data. Three different weather

and geometry scenarios are shown in each row. The column (a) and column (b) show the weather and the geometrical data input to the model. Columns (c) shows the predicted irradiance maps for given inputs in columns (a) and (b). The predicted irradiance maps look almost identical to the reference raytraced simulations in columns (d). The differences are minor and are due to aliasing artifacts as a result of raytracing, which do not appear in the model predictions (see Discussion). It is noteworthy, that for this experiment, the U-Net, optimized by only minimizing the MAE loss, was sufficient to predict the poles that can be seen within the predictions. This is a result of normalizing the data within the range [-1,1] as to the range [0,1] in the experiments (see Experiments in Methods). These results prove the generalizability of the surrogate model to different geometries. Although trained on a limited dataset, it is applicable to other unknown geometries, thereby extending its application in crop and module management.

(a)	(b)
15.05.2021@17:00<br>DNI: 20.37 W/m^2<br>DHI: 90.0 W/m^2<br>Sun altitude: 23.80°<br>Sun azimuth: 271.25°	Pitch: 6.7 m<br>Height: 2.8 m<br>Tilt angle: 0.2°
10.08.2021@12:00<br>DNI: 538.90 W/m^2<br>DHI: 241.94 W/m^2<br>Sun altitude: 54.44°<br>Sun azimuth: 176.87°	Pitch: 9.5 m<br>Height: 3.7 m<br>Tilt angle: -21.4°
03.10.2021@08:00<br>DNI: 0.65 W/m^2<br>DHI: 22.77 W/m^2<br>Sun altitude: 16.24°<br>Sun azimuth: 118.46°	Pitch: 11.6 m<br>Height: 2.5 m<br>Tilt angle: 39.8°

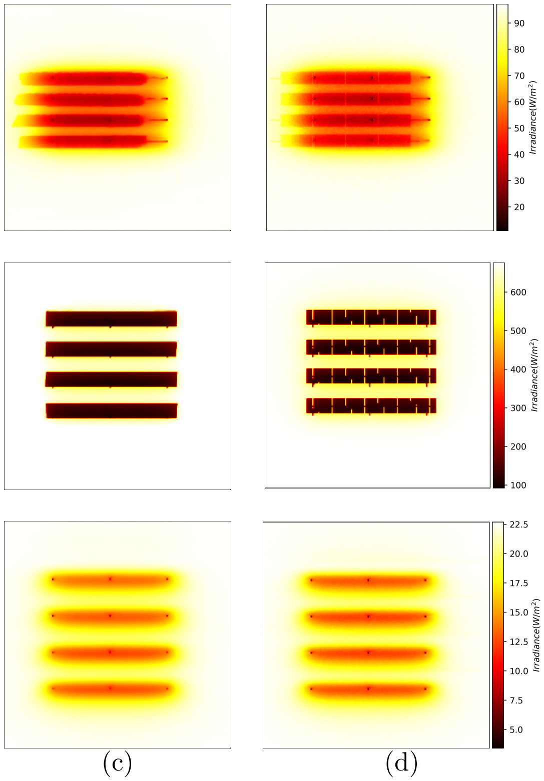

Supplementary Figure 2: Results for different weather scenarios and different geometries in each row: Column (a) reveals the weather input data, while column (b) reveals the geometrical values encoded within the top-view geometry image. The columns (c) and (d) show the predicted irradiance maps and the reference raytracer simulations, respectively.

Supplementary Note 2

Technical details

All experiments and model trainings and tests were conducted on hardware with specifications Intel(R) Xeon(R) CPU E5-2620 v4 @ 2.10GHz and a NVIDIA GPU of 515.43.04 version, which supports CUDA version 11.7 and has a memory capacity of 12GB.

The model used in all the experiments, was implemented within the Pytorch[30] framework.

The computational complexity of our model is characterized by the time required for training and the computational space it occupies. Each training iteration takes approximately 0.06 seconds, and in each epoch, we randomly sample 8,000 datapoints from the dataset, which takes up to 8 minutes to complete. The validation process is more efficient, as each iteration requires less time due to the absence of gradient calculations. During validation, we sample only 1,500 datapoints per epoch, resulting in a quicker evaluation compared to training. Overall, our model takes approximately 14 hours to complete its training. Our model is based on the UNet architecture and comprises 23 convolutional layers, each contributing to the total number of parameters. The overall parameter count is influenced by the kernel size, image size, and the number of features per layer. In our instance, the model consists of approximately 36.9 million parameters and, including the input space, occupies around 765.7 MB of memory. Parameter-intensive convolutional neural networks require to be trained on GPU clusters.

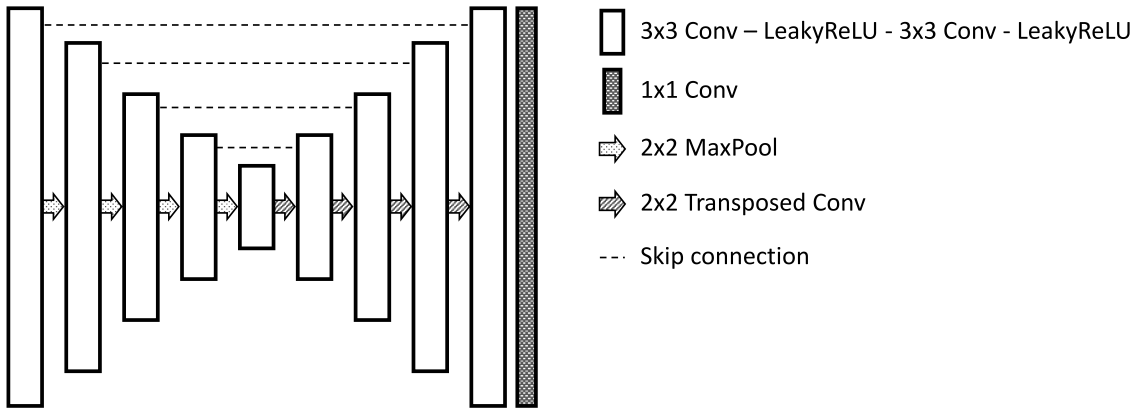

Supplementary Figure 3: Architecture of the surrogate model.
